# Retinopathy in Metabolic Dysfunction-Associated Steatotic Liver Disease

**DOI:** 10.3390/medicina61010038

**Published:** 2024-12-30

**Authors:** Myrsini Orfanidou, Stergios A. Polyzos

**Affiliations:** 1 First Laboratory of Pharmacology, School of Medicine, Aristotle University of Thessaloniki, 54124 Thessaloniki, Greece; 2 First Department of Ophthalmology, AHEPA University Hospital, Aristotle University of Thessaloniki, 54636 Thessaloniki, Greece

**Keywords:** hepatic fibrosis, metabolic dysfunction-associated steatotic liver disease, metabolic dysfunction-associated steatohepatitis, nonalcoholic fatty liver disease, nonalcoholic steatohepatitis, ocular disease, retinopathy

## Abstract

Metabolic dysfunction-associated steatotic liver disease (MASLD) is a multisystemic disease, i.e., influencing various organ systems beyond the liver and, thus, contributing to comorbidities. Characterized by excessive fat accumulation in the hepatocytes, MASLD is frequently linked to metabolic syndrome components, such as obesity, insulin resistance, dyslipidemia, and hypertension. Therefore, exploring the intricate connection between MASLD and other organ systems, including the eyes, seems to be essential. In this context, retinopathy has been investigated for its potential association with MASLD, since both conditions share common pathogenetic pathways. Chronic low-grade inflammation, oxidative stress, insulin resistance, and endothelial dysfunction are only some of those mechanisms contributing to disease progression and, possibly, determining the bidirectional interplay between the liver and retinal pathology. This narrative review aims to summarize data concerning the multisystemicity of MASLD, primarily focusing on its potential association with the eyes and, particularly, retinopathy. Identifying this possible association may emphasize the need for early screening and integrated management approaches that address the liver and eyes as interconnected components within the framework of a systemic disease. Further research is necessary to delineate the precise mechanisms and develop targeted interventions to mitigate the bidirectional impact between the liver and eyes, aiming to reduce the overall burden of disease and improve patient outcomes.

## 1. Introduction

Metabolic dysfunction-associated steatotic liver disease (MASLD) is a global health challenge. It has become the most common chronic liver disease worldwide, and its prevalence displays an increasing trend over the years [1]. Almost a decade ago, MASLD affected about one-quarter of the global population [1]. Nowadays, its prevalence is calculated to be about 30%, whilst specific regions around the world and specific subpopulations are associated with even higher rates [2]. This substantial rise in the prevalence of MASLD is a matter of concern, when considering the limited therapeutic options, since resmetirom, as a specifically targeted medication, has only been approved in the USA, and its high cost may limit its wide availability [3]. MASLD refers to a phenotypic range: it starts as isolated hepatic steatosis that may progress to metabolic dysfunction-associated steatohepatitis (MASH), hepatic fibrosis, cirrhosis, and hepatocellular carcinoma (HCC) [4]. The change in the nomenclature and definition of the disease from nonalcoholic fatty liver disease (NAFLD) to MASLD was suggested by a Delphi consensus in 2023 [5]. This transition from a negative to a positive diagnosis mainly aimed to limit the stigma of the disease and make its association with other metabolic diseases (such as obesity, diabetes mellitus [DM], especially type 2 diabetes mellitus [T2DM], dyslipidemia, arterial hypertension, and cardiovascular diseases [CVD]) clearer [3,4].

MASLD is not a disorder confined within the liver; it has the characteristics of a multisystemic disease [6]. Apart from hepatic-related morbidity and mortality, MASLD is also associated with extrahepatic morbidity and mortality, mainly referring to CVD and neoplasms [7,8]. In this setting, the possibility of an association between MASLD and retinal vascular complications (i.e., retinopathy) has additionally been investigated. This narrative review aims to summarize data concerning the multisystemicity of MASLD, primarily focusing on its potential association with the eyes and, particularly, retinopathy. Identifying this possible association may emphasize the need for early screening and integrated management approaches that address the liver and eyes as interconnected components within the framework of a systemic disease.

## 2. MASLD as a Multisystemic Disease

### 2.1. Pathogenetic Background

The pathogenesis of MASLD has been a field of intense research over the years. The prevailing hypothesis describing its pathogenesis is that of the “multi-hit”, which expands the previously proposed “two-hit” hypothesis, possibly offering a more comprehensive explanation of disease progression [9,10].

#### 2.1.1. The “Two-Hit” Hypothesis: The Initial Conception

The “two-hit” hypothesis initially proposed that the development of MASLD occurs in two phases [11]. During the first phase (first “hit”), insulin resistance leads to increased fat accumulation within the hepatocytes (hepatic steatosis). Subsequent pathogenic contributors (second “hit”), that may be oxidative stress, mitochondrial dysfunction, or pro-inflammatory cytokines, cause inflammation and liver injury, thus leading from hepatic steatosis to steatohepatitis [11]. While this model provided a useful starting point, it became clear that MASLD pathogenesis is more complex, since it may involve multiple intertwined factors. Subsequently, the “multi-hit” hypothesis emerged as a more refined conceptual framework [12,13].

#### 2.1.2. The “Multi-Hit” Hypothesis: A Complex Interaction of Factors

The multi-hit model suggests that MASLD results from the interaction of various genetic, environmental, and metabolic factors that contribute to liver injury. These factors do not necessarily occur sequentially, but often overlap, creating an additive or even a synergistic effect that promotes disease progression [12,13,14].

Insulin resistance is a definite key player in MASLD pathogenesis, especially when considering its association with obesity and T2DM [12,13]. It leads to enhanced lipolysis in the adipose tissue, thereby releasing free fatty acids (FFAs) into circulation, which are taken up by the liver. In turn, the liver increases de novo lipogenesis and reduces fat β-oxidation in the mitochondria, which results in fat accumulation within the hepatocytes. In obese individuals, the expansion of the adipose tissue leads to its dysfunction, thereby altering the secretion of adipokines (e.g., leptin and diponectin) and pro-inflammatory cytokines (e.g., tumor necrosis factor-α [TNF-α] interleukin-6 [IL-6]). This leads to hepatic and systemic inflammation, which exacerbates insulin resistance and promotes further fat accumulation and oxidative stress in the liver [13,15]. Furthermore, accumulated FFAs in the hepatocytes undergo peroxidation, thereby generating reactive oxygen species (ROS). Excessive ROS production overwhelms the antioxidant reserve of the liver, leading to oxidative stress. Mitochondrial dysfunction, which is common in MASLD, plays a central role in the development and progression of oxidative stress by impairing energy metabolism and enhancing ROS production, thus further promoting liver injury and inflammation [13,16].

Moreover, evidence highlights the role of the gut microbiota and the gut-liver axis in the pathogenesis of MASLD. Dysbiosis of gut microbiota increases intestinal permeability, thus allowing endotoxins (e.g., lipopolysaccharides) to enter into the bloodstream and influence the liver, triggering immune responses and hepatic inflammation [12,17]. Gut-derived metabolites, such as short-chain fatty acids and bile acids, also influence hepatic lipid metabolism and inflammation, further adding to MASLD pathogenesis [17]. Chronic oxidative stress, lipotoxicity, and inflammation lead to hepatocellular damage, apoptosis, and the activation of hepatic stellate cells, which are the key contributors to hepatic fibrosis. The immune response, involving both innate and adaptive immunity, contributes to the progression of steatosis to steatohepatitis, fibrosis, and cirrhosis [18,19]. Kupffer cells, which are liver-resident macrophages, and immune cells (e.g., macrophage and monocytes) infiltrating the injured liver, release cytokines that amplify inflammation and fibrogenesis [20,21].

Last but not least, several genes that affect lipid metabolism, inflammation, and/or fibrosis in the liver have been implicated in the pathogenesis of MASLD. Genetic polymorphisms, such as those in the genes *patatin like phospholipase domain containing 3 (PNPLA3)*, *transmembrane 6 superfamily member 2 (TM6SF2)* and *membrane-bound O-acyltransferase 7 (MBOAT7)*, have been linked to increased susceptibility to MASLD and/or advanced disease [12,17]. Epigenetic modifications, including DNA methylation and histone modifications, also play a role in the pathogenesis of MASLD by regulating gene expression in response to environmental factors (e.g., endocrine disruptors) and lifestyle factors (e.g., diet) [22,23].

Taking the above into consideration, it seems that the “multi-hit” hypothesis offers a more integrated understanding of the pathogenesis of MASLD. It emphasizes that MASLD may result from the complex interplay of metabolic, genetic, and environmental factors. It should be highlighted that different pathogenic contributors (“hits”) acting for different durations affect each patient with MASLD; thus, the disease may need individualized diagnosis and management [14].

### 2.2. MASLD and Extrahepatic Diseases

As mentioned above, MASLD is not only a liver-specific disease, but a systemic disease, i.e., it is linked with seemingly diverse extrahepatic diseases. Delving into these connections is crucial for a more holistic management of the patient and for identifying individuals at risk of MASLD-related comorbidities and mortality [24].

CVD is the leading cause of mortality in individuals with MASLD, followed by cancer-related mortality, whereas liver-related mortality lies at the third position [25]. Patients with MASLD are at a higher risk of atherosclerosis, myocardial infarction, and stroke than individuals without MASLD [25]. The underlying mechanisms include chronic low-grade, systemic inflammation, oxidative stress, and endothelial dysfunction, which contribute to accelerated atherogenesis and atheromatous plaque instability [26]. Several studies suggest that MASLD is an independent risk factor of CVD and that its severity is also associated with a higher incidence of cardiovascular events [27]. Chronic kidney disease (CKD) is also strongly associated with MASLD. Patients with MASLD are at higher risk of developing CKD, and this risk is further amplified in the presence of more advanced liver diseases, such as MASLD-associated cirrhosis. Systemic inflammation, insulin resistance, and altered lipid metabolism are central contributors to renal injury and dysfunction [28].

Another substantial association is the one between MASLD and T2DM, which is characterized by the mutual exacerbation of each other [29]. MASLD increases insulin resistance and hepatic glucose output, thereby increasing the risk of developing T2DM or exacerbating existing T2DM. Conversely, T2DM contributes to the development and progression of MASLD to advanced diseases, including steatohepatitis, fibrosis, cirrhosis, and HCC [29]. MASLD has also been linked to other endocrine disorders, including hypothyroidism and growth hormone (GH) deficiency [30,31]. Hypothyroidism can lead to the development of hepatic steatosis, due to lipid dysmetabolism, but also to advanced disease, possibly due to mitochondrial dysfunction, lipid peroxidation, and increased oxidative stress [31]. GH deficiency also contributes to increased visceral obesity and hepatic lipid dysmetabolism, thus promoting the development and possibly the progression of MASLD [30]. Furthermore, women with polycystic ovary syndrome (PCOS) are at a higher risk of developing MASLD, due to shared pathogenetic factors, such as insulin resistance, obesity, and dyslipidemia. The presence of MASLD in patients with PCOS is also associated with higher rates of metabolic syndrome and impaired insulin sensitivity [32].

Obstructive sleep apnea is also highly prevalent in patients with MASLD and is associated with worse liver outcomes. Recurrent hypoxia and disrupted sleep patterns contribute to oxidative stress and systemic inflammation, deteriorating hepatic steatosis, and possibly inflammation and fibrosis [33]. Moreover, the potential associations of MASLD with osteoporosis, sarcopenia, and/or sarcopenic obesity have been supported [34,35,36]. Of interest, patients with MASLD seem to be at a higher risk of fracture compared to individuals without MASLD [35]. Sarcopenia, characterized by low skeletal muscle mass and impaired skeletal function, commonly coexists with MASLD, and a cross-talk between them with potential pathogenic and therapeutic potential has been supported [34,35].

Cognitive impairment, including Alzheimer’s disease and dementia, may also coexist with MASLD [37]. Potential mechanisms interlinking them include systemic inflammation, endothelial dysfunction, and cerebral insulin resistance, potentially leading to neurodegeneration. Additionally, MASLD is associated with higher rates of depression and anxiety [38]. Gastrointestinal diseases, such as irritable bowel syndrome and small intestinal bacterial overgrowth, are associated with MASLD as well. The pathogenesis of both has been partly attributed to altered gut microbiota, increased intestinal permeability, and systemic endotoxemia [39]. Growing interest additionally exists in understanding the link between MASLD and extrahepatic neoplasms. A potential association is suggested mainly concerning gastrointestinal, breast, and gynecological cancer [40]. While evidence is still emerging, chronic inflammation, insulin resistance, as well as glucose and lipid dysmetabolism in MASLD patients may contribute to the development and progression of some types of cancer. Moreover, increasing evidence has been emerging on a potential association between MASLD and autoimmune diseases, such as psoriasis and rheumatoid arthritis [41,42]. While these connections are not yet well-defined, shared inflammatory pathways and systemic immune dysregulation may be potentially common contributors. Of course, a causative association and the potential directionality are hard to show for many of the above-mentioned diseases, since existing studies are mainly observational. Although the existing literature cannot exclude the simple coincidence of some of the above diseases with MASLD, the associations of MASLD with a variety of diverse diseases deserve further clinical and mechanistic research, which may bear diagnostic and therapeutic implications.

## 3. MASLD and Ocular Diseases

### 3.1. The Liver–Eye Axis

The liver–eye axis is an emerging area of research that explores the interconnected pathways through which the liver and eyes influence the function of each other in health and disease. This potentially bidirectional association is part of a broader network of organs interacting through molecular signaling, metabolic regulation, and neural signals. The dysregulation of this network may lead to certain diseases. In this regard, there are studies supporting the association of MASLD with diabetic retinopathy (DR), elevated intraocular pressure (IOP), cataracts, age-related macular degeneration (AMD), dry eye disease, as well as ocular motor cranial nerve palsies.

The association between MASLD and DR is noteworthy, since both conditions are highly prevalent in patients with T2DM and share common risk factors, such as obesity and metabolic syndrome [43,44]. Several studies have focused on investigating a potential link between these two pathologies; however, their results remain conflicting to date. Meta-analyses of observational studies, synthesizing existing data showed no significant association between MASLD and DR overall, compared to individuals without MASLD [45,46,47]. However, some authors supported the distinct association between DR and MASLD within T2DM patients, being positive in some countries (e.g., Italy and India) or inverse in other countries (e.g., China, Korea, and Iran) [45]. Other authors reported that DR showed a positive association specifically with hepatic fibrosis [46], or with MASLD within the subgroup of patients with type 1 diabetes mellitus (T1DM), an association warranting further research to show whether NAFLD may predict retinopathy in T1DM patients [46,47].

Systemic inflammation and vascular dysfunction in MASLD may contribute to elevated IOP and impaired aqueous humor drainage, thereby increasing the risk of glaucoma [48]. In line with this finding, a positive association between MASLD and IOP in Korean adults has been reported [49]. Interestingly, it was also suggested that IOP > 15 mmHg is associated in a dose-dependent manner with more severe MASLD phenotypes [49]. Other authors similarly reported that patients with steatotic liver disease (SLD) had higher rates of elevated IOP (i.e., IOP > 22 mmHg) compared to individuals without SLD [50]. In parallel, the mean IOP of MASLD patients was higher than the mean IOP of controls, whilst elevated IOP was also associated with the severity of SLD in a linear dose–response manner [50].

Furthermore, there is evidence of a potential link between MASLD and the development of cataracts. Although surgical treatment has led to a decline in its prevalence over the last decades, cataract (i.e., lens opacification) still remains the leading cause of blindness in low- and middle-income countries [51]. Several risk factors have been investigated, and a variety of systemic diseases and metabolic disorders have been associated with age-related cataractogenesis, such as T2DM, arterial hypertension, dyslipidemia, and obesity [52], all of which are by definition closely related to MASLD [5]. However, more specifically, it was recently reported that patients with severe MASLD had a higher risk of incident cataract compared to apparently healthy individuals [53]. Other authors suggested that either NAFLD or MAFLD may independently serve as risk factors for the development of cataract [54], as well as another group of authors demonstrated that the higher fatty liver index (FLI), a non-invasive index of hepatic steatosis, was associated with a higher risk of all-cataract, as well as senile-cataract surgery [55].

Another potential association is between MASLD and AMD. AMD is a chronic, degenerative retinal disease that mainly affects the elderly and which may progress to blindness [56]. Data on its association with MASLD are currently limited; however, an interconnection between the two diseases is speculated, which may possibly be mediated through increased systemic oxidative stress and altered the complement activation pathway observed in MASLD [56]. Several genetic loci and variants have been investigated to decode the potential association of MASLD with AMD, some of which are related to the liver (e.g., rs10922109 of complement Factor H; rs10468017 of hepatic lipase), thus enhancing the hypotheses of interaction between MASLD and AMD [57,58].

Last but not least, lipid dysmetabolism and chronic systemic inflammation, which are observed in MASLD, may disrupt the homeostasis of the ocular surface, predisposing individuals to conditions such as dry eye disease [59]. Although this hypothesis may carry value, it has not yet been adequately investigated. Toward this aim, the association of MASLD with hepatic fibrosis and dry eye disease was investigated in patients with primary Sjogren’s syndrome; nonetheless, the results of this study do not confirm a connection, as initially hypothesized [60]. Another intriguing, albeit not well-established association, is that of MASLD with ocular motor nerve palsies (i.e., dysfunction in the cranial nerves responsible for controlling eye movement), which are typically caused by diseases with adverse vascular effects, such as T2DM/T1DM and arterial hypertension, or relevant trauma. Similarly, the metabolic dysregulation observed in MASLD may lead to adverse vascular effects and neuropathy, potentially affecting the cranial nerves, and leading to ocular motor dysfunction [61]. However, further relevant research is needed in order to explore the neurological manifestations of metabolic diseases like MASLD.

### 3.2. Liver–Eye Pathophysiological Associations

The liver and the eyes seem to be linked through a complex network of metabolic, hormonal, and inflammatory signals, highlighting the concept of liver–eye communication. As a central organ in metabolic homeostasis, the liver produces and processes a wide range of biomolecules, including hepatokines, growth factors, and complement proteins, which have profound systemic effects, including on ocular tissues. In this regard, chronic liver diseases, including MASLD, which are increasingly associated with ocular diseases, may contribute to the development and progression of ocular pathology through apparently diverse mechanisms that are summarized hereby [62].

#### 3.2.1. Hepatokines

Hepatokines are liver-secreted biomolecules regarded as substantial in regulating systemic metabolism. They are increasingly recognized for their effect on distant organs, including the eyes [24]. Several hepatokines, including fibroblast growth factor 21 (FGF-21), hepatocyte growth factor (HGF), adropin, and angiopoietin-like proteins (ANGPTLs), have emerged as key factors influencing the liver–eye axis [62].

##### Fibroblast Growth Factor-21

FGF-21 is a hormone-like hepatokine that regulates glucose and lipid metabolism, acting as a systemic mediator of metabolic homeostasis. Secreted primarily by the liver in response to fasting, stress, and fat accumulation, FGF-21 is considered to exert protective effects on peripheral tissues by enhancing insulin sensitivity, reducing lipotoxicity, and suppressing inflammation [63,64]. In the context of the liver–eye interaction, FGF-21 has been reported to act against oxidative stress and inflammation in the retina. Studies suggest that FGF-21 may mitigate retinal damage caused by metabolic insults in conditions such as DR [65]. FGF-21 has been shown to decrease oxidative stress by activating antioxidant pathways and reducing inflammation through the inhibition of the production of pro-inflammatory cytokines [66,67]. The loss of function or the dysfunction of FGF-21, as observed in liver diseases, such as MASLD, may, therefore, lead to an increased susceptibility to vascular dysfunction and retinal damage, contributing to the progression of ocular diseases.

##### Hepatocyte Growth Factor

Another key hepatokine, HGF, is involved in cell growth, tissue regeneration, and angiogenesis. HGF binds to its receptor, c-Met, which is a tyrosine kinase receptor, and activates signaling pathways that promote cell proliferation, survival, and migration [68]. In addition to its effects on liver regeneration and repair, HGF has been implicated in ocular health, particularly in retinal vascular homeostasis [69]. In the eye, HGF helps maintain the integrity of the retinal vasculature by promoting endothelial cell survival and preventing apoptosis [70]. HGF also has anti-inflammatory properties that protect retinal cells from damage caused by chronic metabolic disorders, such as DM [71]. The dysregulation of HGF signaling can contribute to abnormal retinal neovascularization, a hallmark of proliferative DR and wet AMD [71]. Elevated levels of HGF in response to liver injury or inflammation can lead to excessive angiogenesis in the retina, further exacerbating these conditions. Thus, the proper regulation of HGF may be critical for maintaining ocular vascular health, a condition that may be imbalanced in the context of liver-related metabolic diseases.

##### Adropin

Adropin is a hepatokine involved in the regulation of energy homeostasis, lipid metabolism, and endothelial function [72]. Produced primarily by the liver, adropin plays a key role in modulating metabolic pathways that influence both cardiovascular and ocular health. In parallel with the aforementioned hepatokines, adropin is associated with vascular health, as it promotes endothelial function and reduces vascular inflammation [73]. Lower adropin levels, which have been observed in liver diseases, such as MASLD [74], are associated with impaired endothelial function and increased vascular inflammation, both of which can negatively affect the retinal microvasculature [73]. By regulating lipid metabolism and promoting vascular health, adropin seems to serve as a critical link between liver metabolic function and ocular vascular integrity.

##### Angiopoietin-like Proteins

ANGPTLs are a family of secreted proteins with structural similarity to angiopoietins, which are critical regulators of angiogenesis and lipid metabolism [75]. Several members of the ANGPTL family, including ANGPTL3, ANGPTL4, and ANGPTL8, are secreted by the liver and involved in modulating lipid metabolism, inflammation, and vascular homeostasis [76,77]. ANGPTL3 regulates plasma lipid levels by inhibiting lipoprotein lipase, thus controlling triglyceride and cholesterol metabolism [78]. Elevated ANGPTL3 levels, which have been observed in MASLD, can lead to dyslipidemia, which in turn contributes to the progression of retinal diseases characterized by lipid deposition, such as AMD. Additionally, ANGPTL3 has been associated with DR in patients with T2DM [79]. ANGPTL4 is primarily involved in regulating lipid metabolism and vascular permeability [78]. It acts protectively by inhibiting vascular leakage and inflammation [80]. In the eye, ANGPTL4 reduces retinal vascular permeability, which is particularly significant for the prevention of diabetic macular edema. The dysregulation of ANGPTL4 in liver diseases may contribute to increased vascular permeability and exacerbation of retinal edema [81]. ANGPTL8, also known as betatrophin, is involved in glucose and lipid metabolism. It has been implicated in the regulation of triglyceride metabolism, and its dysregulation in liver diseases may exacerbate lipid-related retinal pathologies [82].

#### 3.2.2. Complement System and Inflammatory Mediators

The liver serves as the primary site of production for most complement factors, which are key components of the innate immune system [83]. The dysregulation of the complement system in liver diseases, including MASLD, may affect ocular health [84]. For instance, complement factor H, which is predominantly synthesized in the liver, is considered to be essential in regulating the activation of the complement. Polymorphisms of the complement factor H gene have been associated with AMD [85]. In chronic liver diseases, impaired regulation of the complement system can lead to excessive inflammation and complement-mediated tissue damage, contributing to retinal degeneration and other ocular pathologies [83,86].

Inflammatory mediators, such as C-reactive protein (CRP), IL-6, and TNF-α, which are often elevated in MASLD [87], may also play a critical role in the liver–eye axis. Elevated levels of IL-6 and TNF-α promote inflammation and vascular permeability in the retina [88,89]. In addition, CRP, an acute-phase protein produced by the liver, has been implicated in the pathogenesis of both cardiovascular and ocular diseases, linking systemic inflammation to retinal damage [90].

#### 3.2.3. Insulin Resistance and Lipid Dysmetabolism

Insulin resistance, a central player in the pathogenesis of MASLD [91], leads to hyperglycemia and dyslipidemia, which may adversely affect the metabolism of vascular endothelial cells in the eye, thus contributing to microvascular complications. Such microvascular dysfunction of the retinal vasculature induces or aggravates DR [44].

Furthermore, the liver is central to lipid metabolism; thus, lipid dysmetabolism may affect ocular health. The dysregulation of very-low-density lipoprotein-cholesterol (VLDL-C) and low-density lipoprotein-cholesterol (LDL-C) in MASLD may lead to increased cholesterol and lipid deposition in retinal tissues, contributing to retinal lipid accumulation and drusen formation in AMD [92]. Additionally, the accumulation of FFAs in the liver and the subsequent increase in their circulating levels may trigger lipotoxicity in retinal cells, causing mitochondrial dysfunction and promoting oxidative stress [93].

#### 3.2.4. Oxidative Stress and Mitochondrial Dysfunction

MASLD is associated with increased oxidative stress, which is a key contributor to retinal cell damage. Mitochondrial dysfunction in the hepatocytes leads to excessive production of ROS, which not only affects the liver itself, but also affects distant organs, including the eyes [94]. In the retina, oxidative stress may lead to photoreceptor degeneration, contributing to diseases such as AMD and retinitis pigmentosa [95,96]. The limit in liver capacity to adequately neutralize ROS, due to impaired or exhausted antioxidant defenses, further exacerbates oxidative stress and oxidative-stress endothelial dysfunction, which contributes to several retinal pathological conditions [97,98].

#### 3.2.5. Vitamin A Metabolism and Ocular Health

The liver is the primary storage organ for vitamin A, a vital nutrient for visual function. In liver diseases, such as cirrhosis and cholestasis, impaired vitamin A metabolism may lead to its deficiency that may manifest with ocular symptoms [99]. Vitamin A is essential for the formation of rhodopsin in photoreceptor cells, which is necessary for vision in low-light conditions. Deficiency in vitamin A results in night blindness and, in severe cases, xerophthalmia and corneal ulcers [100].

### 3.3. MASLD and Retinopathy: Current Hypotheses

The potential of an association between MASLD and retinopathy has been under investigation, particularly in the setting of diseases that are clustered under the umbrella of the metabolic syndrome. This conception has emerged owing to the observations that MASLD and retinopathy share common risk factors and underlying pathophysiological mechanisms that may possibly connect liver dysfunction with microvascular complications, including those affecting the retinal vasculature [62]. In this regard, insulin resistance and chronic inflammation seem to play a central role in the association between MASLD and retinopathy [44,62].

Insulin resistance and the related hyperglycemia promote oxidative stress and the formation of glycation end-products, which may break down the blood–retinal barrier, thus adversely affecting retinal capillaries [101]. As MASLD is closely associated with insulin resistance [91], patients with MASLD, particularly those with coexisting DM, are considered to be at higher risk of developing retinopathy [29]. In addition, MASLD has been associated with endothelial dysfunction, which is characterized by reduced nitric oxide bioavailability and increased stiffness of the retinal vasculature [102]. Endothelial damage in the retina may result in reduced blood flow, capillary leakage, and neovascularization, all of which are features of retinopathy [103].

Both MASLD and retinopathy are diseases characterized by chronic, low-grade systemic inflammation. In MASLD, the release of pro-inflammatory cytokines, such as TNF-α and IL-6, is increased [48]. These inflammatory mediators exacerbate endothelial dysfunction, a key feature of microvascular complications, such as DR [103]. Chronic inflammation in MASLD may, therefore, contribute to microvascular damage in the retina.

**Figure 1:**
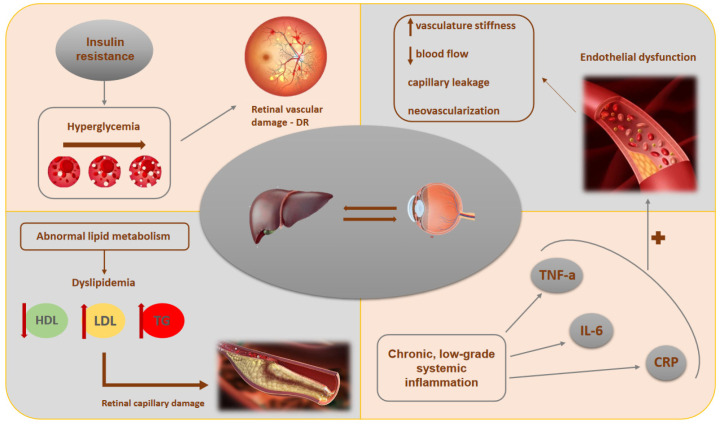
The potential association between MASLD and retinopathy. Insulin resistance and hyperglycemia, which are common in MASLD, promote oxidative stress and glycation end-product formation, thus breaking down the blood–retinal barrier and increasing the risk of retinopathy. MASLD also contributes to endothelial dysfunction, by reducing nitric oxide availability and increasing retinal vascular stiffness, thus leading to features of retinopathy, like capillary leakage and neovascularization. Additionally, chronic inflammation and lipid dysmetabolism exacerbate microvascular damage through pro-inflammatory cytokines, dyslipidemia, and lipid peroxidation, further linking MASLD to retinal disease. Abbreviations: CRP, C-reactive protein; DR, diabetic retinopathy; HDL, high-density lipoprotein; IL-6, interleukin-6; LDL, low-density lipoprotein; MASLD, metabolic dysfunction-associated steatotic liver disease; TG, triglycerides; TNF-a, tumor necrosis factor-a.

In addition, MASLD is characterized by lipid dysmetabolism, which leads to the accumulation of fat in the liver and dyslipidemia (i.e., elevated triglycerides and low high-density lipoprotein-cholesterol [HDL-C]). Dyslipidemia has been implicated in the pathogenesis of retinopathy, as excessive lipids may promote retinal capillary occlusion and damage [104]. Lipid peroxidation and oxidative stress, common in MASLD, may enhance retinal cell damage, thus further linking MASLD to retinal disease [96]. It is of note that fenofibrate, which is a derivative of fenofibric acid approved for the treatment of hypertiglyceridemia, has been suggested to slow the progression of DR, reduce the need for photocoagulation, as well as the macula volume in patients with diabetic macular edema [105,106,107]. Given the strong association between MASLD and hypertiglyceridemia, the potential role of fenofibric acid or its derivatives (e.g., fenofibrate) in mitigating retinopathy in the setting of MASLD is particularly relevant, but warrants further research. Figure 1 summarizes the current hypotheses concerning the potential association between MASLD and retinopathy.

### 3.4. MASLD and Retinopathy: Data from Clinical Studies

During the last two decades, several observational clinical studies evaluating the association between MASLD and retinopathy have been published. Mainly, they are cross-sectional [108,109,110,111,112,113,114,115,116,117,118,119,120,121,122,123,124,125,126,127,128,129,130,131,132], but there are also four case–control studies [133,134,135,136] and four cohort studies [137,138,139,140]. Importantly, most studies are based on the definition of NAFLD, instead of the recently recommended definition of “MASLD”, with the exception of two studies [132,140]. Therefore, we hereby decided to keep the definition of NAFLD, because NAFLD, MAFLD, and MASLD are very similar entities, but they may not be used interchangeably [141]. One of the studies with the MASLD population investigated the association between retinal atherosclerosis and both NAFLD and MAFLD [140], while the other investigated the association of MASLD with retinopathy [132]. The main characteristics and outcomes of all the above-mentioned studies are summarized in Table 1. Most, but not all, reported higher rates of retinopathy in patients with NAFLD [108,110,111,113,115,117,119,120,122,124,125,129,131,132,136,139]. However, there are also studies that resulted in no statistically significant association [109,112,118,121,123,127,134,135,137,138,140], as well as fewer studies that reported lower rates of retinal pathology in NAFLD than non-NAFLD controls [114,116,126,128,130,133].

It is noteworthy that almost all studies included patients with DM, mainly T2DM. Taking into account that dysglycemia and insulin resistance are considered to be pathogenetic mechanisms implicated in both MASLD and retinopathy, it is rational that relevant research has included relevant populations. On the other hand, the inclusion of patients with DM in these studies is a highly probable confounding factor. When looking into the type of DM, the results of studies with NAFLD/MASLD and T2DM were rather conflicting [108,109,111,112,114,116,117,120,121,123,126,129,130,131,133,134,135,136,137,138]. On the contrary, all studies that included patients with NAFLD/MASLD and T1DM reported higher rates of retinopathy in patients than controls [110,113,115,124,132]. This observation may imply that the parameter of disease duration might also be important in this association, given that T1DM usually has a longer duration than T2DM, and so the effect of the relevant dysglycemia on both the liver and the retina may be longer in studies with T1DM than those with T2DM [47]. The interrelationship between MASLD and T1DM has only recently been highlighted and further research is needed to explore the potential impact of T1DM and its duration on the association between MASLD and retinopathy; in other words, we need well-designed studies to clarify whether MASLD is associated with retinopathy independently of the presence and the duration of T1DM. Moreover, it is of interest that some studies that included both patients with and without DM also reported higher rates of retinopathy in NAFLD than the control group [119,139]. Furthermore, patients with DM or arterial hypertension were excluded in another study, supporting the association of NAFLD with retinopathy after the elimination of two potential confounding factors of retinopathy [125].

As far as the diagnosis of NAFLD is concerned, it was established with imaging in most studies reporting on the association between NAFLD/MASLD and retinopathy. Specifically, abdominal ultrasonography was the main diagnostic method used [108,109,110,111,112,113,114,115,116,117,118,119,121,122,123,124,125,126,128,130,131,133,134,136,137,138], although transient elastography is also reported in five studies [121,123,127,129,135], and magnetic resonance imaging (MRI) in one study [134]. Non-invasive indices of hepatic steatosis and/or fibrosis have also been used in a few studies [120,131,132]. No distinct pattern of association is observed, which was based on the diagnostic method of NAFLD; however, this should carefully be interpreted because of the very small number of studies with MRI or other imaging techniques that are more sensitive for hepatic steatosis than ultrasonography. Importantly, to date, no relevant study of NAFLD diagnosis is based on hepatic histology after liver biopsy. Thus, potential differences in the association of NAFLD/MASLD with retinopathy according to different histological lesions (e.g., steatosis, hepatocellular ballooning, and, more importantly, fibrosis) were not to date investigated.

All the above considered, there is a need for further research in the field, especially with specifically designed prospective cohort studies, which may strengthen the association between MASLD and retinopathy or may reject it. As mentioned above, to date, there are four relevant cohort studies, the main characteristics of which are summarized in Table 1. The first of them was published in 2019 [137], and the other three in 2023 [138,139,140]. Overall, three studies reported no significant association between MASLD and retinopathy [137,138,140], whereas one reported that patients with newly diagnosed NAFLD were associated with increased incidents of retinopathy [139]. This study included a substantially large population; however, data were collected retrospectively from a registry, and the diagnoses of both NAFLD and retinopathy were based on ICD-10 codes, which bears the possibility of bias [139]. Another of these studies stratified NAFLD according to the severity of the disease (i.e., mild, moderate, and severe) based on ultrasonographical criteria [137]. Both moderate and severe NAFLD were associated with lower rates of retinopathy compared to controls, whilst mild disease phenotype displayed no significant association. Although this study provided inverse than expected results, it also supported the finding that the association between MASLD and retinopathy may be different according to the severity of the liver disease [137]. Interestingly, another of these cohort studies, which focused on retinal atherosclerosis, supported that it was associated with MAFLD, but not NAFLD [140]. This further supports what was mentioned above [141], i.e., that NAFLD, MAFLD, and MASLD are similar entities, but these terms cannot be used interchangeably. NAFLD-related findings should be cautiously extrapolated to patients with MAFLD or MASLD.

Taking all the above into consideration, the results of clinical studies on the association between MASLD and retinopathy remain inconclusive to date, partly owing to the design of existing relevant studies, being observational and, therefore, being possibly affected by residual confounding factors. Future studies in the field may possibly focus on the association of MASLD with retinopathy in patients with T1DM; since existing studies have provided evidence for a potential association, mechanistic studies are warranted to shed light on the potential underlying mechanisms. More longitudinal studies focusing on the duration and severity of MASLD are also needed, as well as studies with histological endpoints.

## 4. Conclusion

The potential association between MASLD and retinopathy highlights the need for a comprehensive approach to understanding and managing MASLD as a multisystemic disease. The shared pathophysiological mechanisms point to a complex interplay which underscores that the liver cannot be viewed as an isolated organ, but in a setting of interconnections with other organs, possibly including the eyes. In this regard, liver diseases, including MASLD, cannot be managed without considering this functional interconnection of the liver with other organs, but also without considering the effects of the potentially used drugs on this interconnection. This insight reinforces the importance of early detection and interdisciplinary collaboration to manage patients at risk of developing MASLD and ocular diseases. In the context of their potential association, regular ophthalmologic screenings should be prioritized for MASLD patients, especially those with concomitant DM or metabolic syndrome. Effective management of metabolic risk factors, as well as lifestyle interventions, may improve both liver and eye health, although it remains to be specifically shown. Collaboration between hepatologists, endocrinologists, and ophthalmologists seems to be crucial for providing holistic care to these patients.

All the above considered, specifically designed mechanistic studies are warranted to provide valuable information toward the potential causality between MASLD and retinopathy. Thus, future research should focus on elucidating the molecular and cellular pathways potentially linking MASLD and retinopathy, as well as other ocular diseases, in order to shed light on their potentially causal association or, inversely, to prove that this association is only an epiphenomenon, i.e., it is driven by other parameters (e.g., DM) rather than MASLD itself. In clinical terms, the causality between MASLD and retinopathy is hard to show, since this requires the setting of randomized controlled trials, which meet obvious ethical considerations. However, specifically designed prospective cohort studies may, hopefully, provide the best evidence toward this aim, i.e., they may strengthen the association between MASLD and retinopathy, but again, they cannot show causality. Additionally, clinical trials on investigational drugs for MASLD may take into consideration the potential liver–eye axis, but also the potentially adverse effects of these medications on the eyes, which seems to be currently underrecognized. Expanding our understanding of the liver–eye axis may ultimately improve patients’ outcomes by enabling more precise preventive and therapeutic measures to address the root of metabolic causes affecting both the liver and ocular health.

## Figures and Tables

**Table 1:** Main characteristics and outcomes of observational clinical studies on the association between MASLD and retinopathy.

First Author, Year, Origin (Reference) *	Study Design	Study Groups (n)	Main Findings
Targher, 2008, Italy [108]	Cross-sectional	Patients with T2DM and US-diagnosed NAFLD (1421) vs. patients with T2DM without NAFLD (682).	NAFLD was associated with higher rates of both NPDR and PDR/la ser-treated retinopathy.
Leite, 2009, Brazil [109]	Cross-sectional	Patients with T2DM and US-diagnosed NAFLD (125) vs. patients with T2DM without NAFLD (55).	NAFLD was not associated with retinopathy.
Viswanathan, 2010, India [136]	Case–control	Patients with T2DM and US-diagnosed NAFLD (156) vs. patients with T2DM without NAFLD (142).	NAFLD was associated with higher rates of NPDR but not PDR.
Targher, 2010, Italy [110]	Cross-sectional	Patients with T1DM and US-diagnosed NAFLD (111) vs. patients with T1DM without NAFLD (91).	NAFLD was associated with higher rates of retinopathy.
Mantovani, 2012, Italy [111]	Cross-sectional	Patients with T2DM, hypertension and US-diagnosed NAFLD (59) vs. patients with T2DM and hypertension without NAFLD (57).	NAFLD was associated with higher rates of retinopathy.
Takeuchi, 2012, Japan [112]	Cross-sectional	Patients with T2DM and US-diagnosed NAFLD (77) vs. patients with T2DM without NAFLD (169).	NAFLD was not associated with retinopathy.
Yoneda, 2012, Japan [113]	Cross-sectional	Patients with T1DM and US-diagnosed NAFLD (25) vs. patients with T1DM without NAFLD (119).	NAFLD was associated with higher rates of retinopathy.
Lv, 2013, China [114]	Cross-sectional	Patients with T2DM and US-diagnosed NAFLD (742) vs. patients with T2DM without NAFLD (475).	NAFLD was associated with lower rates of retinopathy. This result persisted even after stratification according to sex.
Vendhan, 2014, India [115]	Cross-sectional	Patients with T1DM and US-diagnosed NAFLD (204) vs. patients with T1DM without NAFLD (536).	NAFLD was associated with higher rates of retinopathy. This result persisted after adjustment for sex, DM duration, over weight/obesity, hypertension, fasting plasma glucose, and nephropathy.
Kim, 2014, Korea [116]	Cross-sectional	Patients with T2DM and US-diagnosed NAFLD (588) vs. patients with T2DM without NAFLD (341).	NAFLD was associated with lower rates of both NPDR and PDR. This result persisted after adjustment for age, sex, HbA1c, C-peptide, DM duration, BMI, and hypertension.
Somalwar, 2014, India [117]	Cross-sectional	Patients with T2DM and US-diagnosed NAFLD (68) vs. patients with T2DM without NAFLD (52).	NAFLD was associated with higher rates of retinopathy.
Yang, 2015, China [119]	Cross-sectional	Patients with US-diagnosed NAFLD (872) vs. patients without NAFLD (1582).	NAFLD was associated with higher rates of retinopathy.
Lin, 2016, Taiwan [118]	Cross-sectional	Patients with DM and US-diagnosed NAFLD (459) vs. patients with DM without NAFLD (486) vs. patients with NAFLD without DM (1211) vs. controls without either DM or NAFLD (3807).	NAFLD in patients with DM was associated with higher rates of retinopathy compared to controls, but not associated with retinopathy compared to patients with DM without NAFLD. NAFLD in patients without DM was not associated with retinopathy compared to controls. These results persisted after adjustment for age, gender, ethnicity, WC, serum HDL-C, serum TGs, SBP, and HbA1c
Yan, 2016, China [126]	Cross-sectional	Patients with T2DM without NAFLD (69) vs. patients with T2DM and US-diagnosed NAFLD (T2DM longer duration than NAFLD; 62) vs. patients with T2DM and US-diagnosed NAFLD (NAFLD longer duration than T2DM; 81).	NAFLD in patients with shorter duration of T2DM was associated with lower rates of retinopathy compared to both other groups.
Mantovani, 2017, Italy [122]	Cross-sectional	Patients with T1DM and US-diagnosed NAFLD (150) vs. patients with T1DM without NAFLD (136).	NAFLD was associated with higher rates of retinopathy.
Romero-Ibarguengoitia, 2017, USA [125]	Cross-sectional	Patients with US-diagnosed NAFLD without DM and hypertension (40) vs. individuals without NAFLD, DM or hypertension (112).	NAFLD was associated with higher rates of retinopathy.
Mantovani, 2019, Italy [123]	Cross-sectional	Post-menopausal women with T2DM and NAFLD—US-diagnosed steatosis; TE-defined fibrosis (10) vs. post-menopausal women with T2DM with steatosis alone (52) vs. post-menopausal women with T2DM without steatosis (15).	NAFLD was not associated with retinopathy.
Zhang, 2019, China [137]	Cohort	Patients with T2DM and US-diagnosed NAFLD (254) vs. patients with T2DM without NAFLD (161).	Overall, NAFLD was not associated with retinopathy. Moderate and severe NAFLD was associated with lower rates of retinopathy compared to controls. Mild NAFLD was not associated with retinopathy.
Afarideh, 2019, Iran [133]	Case–control	Patients with T2DM and US-diagnosed NAFLD (248) vs. patients with T2DM without NAFLD (687).	NAFLD was associated with lower rates of retinopathy. This result persisted after the adjustment for BMI, WC, and SBP but turned to statistically non-significant after including more potential covariates.
Ciardullo, 2020, Italy [120]	Cross-sectional	Patients with T2DM and NAFLD based on several noninvasive biomarkers vs. patients with T2DM without NAFLD (total n: 2770).	NAFLD was associated with higher rates of retinopathy in patients with intermediate and a high AST/ALT ratio.
Lombardi, 2020, Italy [121]	Cross-sectional	Patients with T2DM and US-diagnosed and TE-diagnosed NAFLD (351/171) vs. patients with T2DM without NAFLD (43/67) CAP measurements available only in 238 of 394 patients.	Based on US diagnosis, NAFLD was not associated with retinopathy. Based on CAP, NAFLD was not associated with retinopathy. Based on LSM, NAFLD was associated with higher rates of retinopathy in patients with higher rates of presumed fibrosis.
Popa, 2020, Romania [124]	Cross-sectional	Patients with T1DM and US-diagnosed NAFLD (55) vs. patients with T1DM without NAFLD (126). Patients with T1DM and HSI ≥ 36 (72) vs. patients with T1DM and HIS < 30 (126). Patients with T1DM and FLI ≥ 60 (80) vs. patients with T1DM and FLI < 30 (48).	Based on US diagnosis, NAFLD was associated with higher rates of retinopathy. Based on HIS, NAFLD was associated with higher rates of retinopathy. Based on FLI, NAFLD was associated with higher rates of retinopathy.
Sawadjaan, 2020, Philippines [127]	Cross-sectional	Patients with DM and TE-diagnosed NAFLD (39) vs. patients with DM without NAFLD (45).	NAFLD was not associated with retinopathy.
Mikolasevic, 2021, Croatia [129]	Cross-sectional	Patients with T2DM and TE-diagnosed NAFLD (372) vs. patients with T2DM without NAFLD (70).	NAFLD (both steatosis and fibrosis) was associated with higher rates of retinopathy.
Rivera-Esteban, 2022, Spain [135]	Case–control	Patients with T2DM and TE-diagnosed NAFLD (124) vs. patients with T2DM without NAFLD (62).	NAFLD was not associated with retinopathy.
Hermans, 2022, Belgium [128]	Cross-sectional	Patients with DM, AD and US-diagnosed NAFLD (272) vs. patients with DM and AD without NAFLD (66) vs. patients with DM and NAFLD without AD (235) vs. patients-controls with DM, without AD and NAFLD (171).	NAFLD was associated with lower rates of retinopathy in both groups with and without AD compared to controls.
Wen, 2022, China [130]	Cross-sectional	Patients with T2DM and US-diagnosed NAFLD (1181) vs. patients with T2DM without NAFLD (747).	NAFLD was associated with lower rates of retinopathy. This result persisted after all adjustments.
Ren, 2023, China [134]	Case–control	Patients with T2DM and MRI (IDEAL-IQ sequence)-diagnosed NAFLD (41) vs. patients with T2DM without NAFLD (40).	NAFLD was not associated with retinopathy.
Wang, 2023, China [140]	Cohort	Patients with MAFLD and/or NAFLD (na) vs. individuals without MAFLD or NAFLD (na)	MAFLD was associated with higher rates of retinal atherosclerosis. NAFLD was not associated with retinal atherosclerosis.
Deravi, 2023, Iran [138]	Cohort	Patients with T2DM and US-diagnosed NAFLD (1215) vs. patients with T2DM without NAFLD (1908).	NAFLD was not associated with retinopathy.
Ebert, 2023, Sweden [139]	Cohort	Patients with newly diagnosed NAFLD based on registered ICD-10 codes (6785) vs. individuals without NAFLD (61136)	NAFLD was associated with higher incident retinopathy. This result persisted after all adjustments and after stratification according to follow-up timepoint, sex and age.
Erman, 2024, Turkey [131]	Cross-sectional	Patients with T2DM and US-diagnosed NAFLD (na) vs. patients with T2DM without NAFLD (na). Fibrosis estimated by noninvasive scores (FIB-4, NFS)	FIB-4 and NFS scores were associated with higher rates of retinopathy. This result persisted after adjustment for age, sex, HbA1c, and DM duration.
Mantovani, 2024, Italy [132]	Cross-sectional	Patients with T1DM and MASLD (HSI > 36; 578) vs. patients with T1DM and MASLD with fibrosis (FIB4 ≥ 1.3; 93) vs. patients without MASLD (738).	MASLD with or without significant fibrosis was associated with higher rates of retinopathy compared to controls. MASLD with significant fibrosis was associated with higher rates of retinopathy compared to MASLD without fibrosis.

*: Studies are sorted according to the year of publication. Abbreviations: AD, atherogenic dyslipidemia; ALT, alanine aminotransferase; AST, aspartate aminotransferase; BMI, body mass index; CAP, controlled attenuation parameter; DM, diabetes mellitus; DR, diabetic retinopathy; FIB-4, fibrosis-4 index; FLI, fatty liver index; HbA1c, hemoglobin A1c; HDL-C, high-density lipoprotein-cholesterol; HSI, hepatic steatosis index; ICD-10, international classification of diseases-tenth revision; LSM, liver stiffness measurement; MRI, magnetic resonance imaging; NAFLD, nonalcoholic fatty liver disease; NFS, NAFLD fibrosis score; NPDR, non-proliferative diabetic retinopathy; PDR, proliferative diabetic retinopathy; SBP, systolic blood pressure; RVC, retinal vascular changes; PPV, positive predictive value; NPV, negative predictive value; T1DM, type 1 diabetes mellitus; T2DM, type 2 diabetes mellitus; TE, transient elastography; TGs, triglycerides; US, ultrasonography; WC, waist circumference.

## Data Availability

No raw data were collected, since this is a narrative review.
